# Self-report questionnaires, behavioral assessment tasks, and an implicit behavior measure: do they predict social anxiety in everyday life?

**DOI:** 10.7717/peerj.5441

**Published:** 2018-08-10

**Authors:** Isabel L. Kampmann, Paul M.G. Emmelkamp, Nexhmedin Morina

**Affiliations:** 1 Department of Clinical Psychology, University of Amsterdam, Amsterdam, the Netherlands; 2 Institute of Psychology, Westfälische Wilhelms-Universität Münster, Münster, Germany; 3 HSK Groep Woerden, Woerden, Netherlands

**Keywords:** Behavioral assessment task, Self-report questionnaire, Approach-avoidance task, Social anxiety, Virtual reality

## Abstract

Social anxiety is commonly assessed with self-report measures. This study aimed to investigate whether maximum anxiety levels during in vivo and virtual reality behavioral assessment tasks (BATs), and implicit approach-avoidance tendencies during the approach-avoidance task (AAT) explain more variation as predictors of daily social anxiety than self-report measures. A total of 62 university students (*M*_age_ = 20.79; *SD* = 4.91) with high levels of social anxiety completed self-report measures on fear of negative evaluation (FNE-B) as well as fear and avoidance in social situations (Liebowitz social anxiety scale-self report), in vivo and virtual reality BATs, and the AAT (independent variables) in the laboratory. On seven consecutive days, social anxiety, experiential avoidance, and negative social events (dependent variables) were assessed. The results revealed that fear of negative evaluation predicted everyday social anxiety and experiential avoidance. Fear and avoidance in social situations only predicted experiential avoidance. Neither implicit approach-avoidance tendencies during the AAT nor maximum anxiety levels during the in vivo and virtual reality BATs predicted any outcome variable. Our results support the use of self-report questionnaires in the assessment of social anxiety.

## Introduction

In research and clinical practice, social anxiety, the fear of social situations in which one might behave embarrassingly, be scrutinized, and negatively evaluated, is commonly assessed using self-report measures. The application of self-report questionnaires, such as the Liebowitz social anxiety scale-self report (LSAS-SR; Liebowitz, 1987) that measures fear and avoidance in social situations and the fear of negative evaluation scale (FNE-B; Leary, 1983), assessing fear of being negatively evaluated in social situations, has the advantage of high time- and cost-effectiveness. However, self-report questionnaires are prone to recall bias and the reliance on self-report measures, rather than the measurement of observable behavior, has been criticized (Baumeister, Vohs & Funder, 2007; Furr, 2009).

An alternative to asking participants to recall fear in past social situations is to directly assess anxiety in social situations. A standardized behavioral assessment task (BAT) that has been used in previous research on social anxiety (Amir et al., 2008) is the impromptu speech paradigm (Beidel et al., 1989). Here, individuals are instructed to give a speech for a certain period of time in front of a camera and/or audience after a short preparation period on one of several preselected topics. It can be hypothesized that social anxiety reported in a social situation, such as a BAT, is a better predictor of social anxiety in daily life than self-report measures.

A limitation of the speech paradigm might be however, that it targets only public speaking anxiety which, although very common (Ruscio et al., 2008), does not necessarily trigger social anxiety in all individuals. Furthermore, some individuals with public speaking anxiety might not experience social anxiety in other social situations, which poses limitations in the extent to which the speech paradigm may predict social behavior in other situations. However, the standardized simulation of other social situations in which social anxiety can be reliably assessed is rather difficult in real life. A solution might be to simulate social situations using virtual reality technology. By this means, diverse social situations, including verbal interactions, can be simulated. A BAT using virtual social scenarios would allow the assessment of social anxiety in highly standardized situations. Previous research has shown that virtual reality environments that resemble feared social interactions in real life can effectively be used to treat individuals with social anxiety disorder (Bouchard et al., 2011; Kampmann et al., 2016). Moreover, effects of exposure to virtual environments can be measured in real life (Morina et al., 2015). Accordingly, a standardized virtual reality BAT that does not require the presence of humans might be useful in assessing social anxiety in different social situations. However, to our knowledge, the validity of using virtual reality in assessing social anxiety has not been examined yet and it is hence relevant to investigate, whether anxiety in a BAT using virtual reality can predict social anxiety in real life.

Social anxiety is often accompanied by avoidance behavior which can contribute to the maintenance of the anxiety (Clark & Wells, 1995). As with anxiety, the LSAS-SR measure assesses avoidance of social situations in the past. Measuring avoidance behavior directly might lead to more reliable information on individual’s social anxiety and avoidance in daily life by circumventing recall bias. An assessment tool that measures implicit approach- and avoidance tendencies is the approach-avoidance task (AAT; Rinck & Becker, 2007). By assessing reaction times (RTs) for pull and push movements with a joystick in response to social anxiety-related stimuli (e.g., faces), the AAT measures automatic action tendencies. Accumulative research suggests that social anxiety affects early perception processes for social stimuli, particularly angry faces (Staugaard, 2010), which has also been reflected in increased implicit avoidance tendencies for angry and happy faces during the AAT (Heuer, Rinck & Becker, 2007; Roelofs et al., 2010). As an assessment task, the AAT would allow to measure implicit behavior in a highly standardized way and one can hypothesize that approach-avoidance behavior, as measured with the AAT, might predict social anxiety in daily life.

The aim of the present study was to assess whether in vivo and virtual reality BATs and an AAT explain more variance as predictors of social anxiety in daily life than self-reports measures. To do so, we tested whether two commonly used self-report questionnaires, a BAT containing a speech, a BAT using virtual situations, and approach-avoidance behavior assessed with the AAT, predicted social anxiety in everyday life. Moreover, we were interested whether the described measures predict experiential avoidance, referring to the tendency to control and extinguish unpleasant internal events, such as negative emotions, thoughts, and bodily sensations (Hayes et al., 1996), which has been associated with social anxiety (Kashdan et al., 2014; Kashdan, Weeks & Savostyanova, 2011) and the experience of negative social events in daily life.

## Methods

### Participants

All first year psychology students of the University of Amsterdam in 2014 were asked to fill in a number of questionnaires including the social interaction anxiety scale (Mattick & Clarke, 1998) as an obligatory part of the first year of their study. Individuals belonging to the 50% of those with the highest social anxiety scores were invited to participate and screened for exclusion criteria. The exclusion criteria were current pregnancy, current use of tranquilizers, medication, and/or drug use at the time of testing, and being younger than 18 years. This resulted in 62 participants (50 female) ranging in age from 18 to 46 years (*M* = 20.79; *SD* = 4.91). Participation was compensated with partial course credit.

### Independent variables

#### Self-report measures

Fear and avoidance in social situations were measured using the Dutch self-report version of the LSAS-SR (Liebowitz, 1987). This 24-item questionnaire is rated on a 4-point Likert scale with higher scores indicating higher levels of social anxiety. The LSAS-SR has a high 12-week test–retest reliability (*r* = 0.83; Baker et al., 2002) and also a high internal consistency in the present study (Cronbach’s *α* = 0.92).

Fear of negative evaluation in social situations was assessed by means of the Dutch version of the fear of negative evaluation scale-brief form (FNE-B; Leary, 1983). This self-report questionnaire contains 12 items rated on a 5-point Likert scale with higher scores implying higher fear of negative evaluation. Psychometric properties have been reported to be good in earlier research (Weeks et al., 2005) and the internal consistency in the present study was high (Cronbach’s *α* = 0.95).

#### Approach-avoidance task

Implicit approach-avoidance tendencies were assessed using the AAT (Rinck & Becker, 2007). In this task, pictures of female and male faces with neutral, angry, happy, and disgust expressions, from the Karolinska directed emotional faces (Lundqvist, Flykt & Öhman, 1998), were presented on a computer screen in a random order. Starting with a joystick in a middle position, participants were instructed to make a pull- or push movement with the joystick depending on the color of the picture that was presented rather than the facial image. When the hue was a sepia tone, participants pushed the joystick away from themselves toward the computer screen. When the hue was a greyscale tone, participants pulled the joystick toward themselves away from the computer screen. During pull movements, the size of the picture on the screen increased (approach condition) while the size decreased until the picture disappeared during push movements (avoidance condition). In the 16 practice trials and 80 assessment trials, the number of approach and avoidance trials was equal.

Prior to the calculation of mean RTs for the eight combinations of facial expression (neutral, angry, happy, and disgusted) and movement (push and pull), all incorrect trials (5.7%) and trials with RTs that deviated more than 3 *SD*s from the participant’s mean RT (1.6%), were dismissed. RTs reflect the time needed to complete the movement. To determine an approach-avoidance bias score, we subtracted the participant’s mean RTs of all pull trials from the mean RT of all push trials for each picture type (Heuer, Rinck & Becker, 2007). Stronger approach than avoidance tendencies are represented by bias scores with positive values, while stronger avoidance than approach tendencies are indicated by bias scores with negative values. Given that social anxiety seems to be associated with increased vigilance and avoidance of threatening faces (Chen & Clarke, 2017; Staugaard, 2010) and to preserve statistical power, only approach-avoidance bias for angry and disgusted faces were included in further analyses.

#### Virtual reality behavioral assessment tasks

The virtual reality BAT consisted of two virtual situations in which participants engaged in conversation with virtual humans. For this purpose, participants put on a head mounted display (HMD; Sony HMZ-T2 with a custom-built head position tracker) which was connected to a laptop (Dell Inspiron 7720 laptop running Windows 7 64 bits) that generated the virtual situations. Dialogues between virtual humans and participants were automated. That is, the system detected participants’ answers via a microphone by means of key word recognition and speech detection technology (Hartanto et al., 2016). Dialogues were prewritten and therefore, the virtual human could not respond to the specific content of the participant’s answer. However, short answers referring to the participant’s answer were used (e.g., “Ah, I see!,” “Ok, thank you”) in order to give the participant the feeling that the virtual human responded to his/her answer. Before entering a virtual situation, participants saw a written instruction via the HMD explaining what they had to do during the exercise. In the first virtual situation, participants were standing at a bus stop when a stranger walked up to them and asked multiple questions (i.e., about the bus ticket system in the Netherlands). In the second virtual situation, participants attended a foreign language class (i.e., English) in which a teacher asked the attendees random questions (e.g., “Which superpower would you like to have?”) one by one (Qu et al., 2015). Given that in the in vivo BAT topics were used that were not related to the personal life of the participants, we also chose to ask a non-personal question in the virtual reality BAT. These scenarios were selected because they target two very common social fears, the fear of speaking in front of others and the fear of small talk with unknown people. Participants perceived the virtual scenarios in first person perspective. The duration of both virtual situations was 5 min. The experimenter was not present during the virtual reality BAT because we wanted to investigate the social anxiety elicited by the virtual humans. The presence of a real person could have led to increased social anxiety which could have confounded the results.

Anxiety during the virtual reality BAT was assessed using subjective units of distress (SUD; Wolpe, 1969). After each virtual situation, participants were asked to rate the highest level of anxiety they had experienced during the virtual situation on a scale from 0 (not anxious) to 10 (very anxious). Correlational analyses revealed that the SUD scores of the two virtual worlds significantly correlated (*r* = 0.54). Therefore, a mean score was calculated which resulted in one anxiety score (virtual reality SUD) for each participant.

#### In vivo behavioral assessment tasks

The in vivo BAT comprised a modified impromptu speech task (Beidel et al., 1989) in the presence of the experimenter. Participants were told that they would need to give a 5 min speech in front of a camera and that their speech performance would be rated afterward by independent raters. They were asked to choose one out of five topics (nuclear power, euthanasia, republic or monarchy, burqa ban, mandatory organ donation) and they had 2 min to prepare the speech (Kampmann et al., 2016). During preparation, taking notes was allowed but participants were told that they were not allowed to use the notes during the speech. During the speech, participants were standing in front of a camera and they were instructed to look at the camera while speaking. The duration of the speech was 5 min or until participants expressed that they wanted to stop. Similar to the virtual reality BAT, participants rated the highest level of anxiety they had experienced during the speech using SUDs on a scale from 0 (not anxious) to 10 (very anxious) after the speech (in vivo SUD).

### Dependent variables

Social anxiety in daily life activities was assessed with a Dutch translation of the state social anxiety questionnaire (SSA; Kashdan & Steger, 2006), a seven-item measure with a 5-point Likert scale with higher scores indicating higher levels of everyday social anxiety (e.g., “I found it hard to interact with people”). The items of this scale were modified items from other scales (see Kashdan & Steger, 2006). The SSA score for each participant was derived by calculating the mean of the SSA scores of the 7 days. The internal consistency in the present study was high (Cronbach’s *α* = 0.88–0.93).

For the assessment of experiential avoidance, we used the four-item state measure of experiential avoidance (SMEA) developed by Kashdan et al. (2014) translated into Dutch: “How much did you try to hide and/or conceal your anxiety from others?,” “To what degree did you give up saying or doing what you like (or mattered to you) in order to control and manage anxiety?,” “How much did you try to control your anxiety-related feelings or thoughts?,” and “How upset and bothered were you about anxiety-related feelings or thoughts?.” All items were rated on a 7-point Likert scale ranging from 1 (not at all) until 7 (very much) at the following 7 days. By calculating the mean of the seven SMEA scores, we received one SMEA score for each participant. The internal consistency in the present study was high (Cronbach’s *α* = 0.84–0.92).

Negative social situations experienced during the day were assessed with a modified version of the daily event survey (DES; Butler, Hokanson & Flynn, 1994) translated into Dutch. We used 13 items related to social events that were developed for student populations and of which six items were on negative social events (e.g., “Did something awkward or embarrassing in a social situation.”) and seven items on positive social events (e.g., “Had especially good interactions with friend(s) or acquaintances.”). To calculate a DES score, we averaged the number of events of the 7 days for positive and negative events separately (Zeigler-Hill, Myers & Clark, 2010). Then, we divided the mean negative event score by the total number of events (mean negative plus mean positive events) multiplied by 100, to receive the percentage of negative social events experienced during the day. The Dutch versions of the SSA, SMEA, and DES were translated from the originals by a native Dutch speaker and retranslated by a native English speaker.

### Procedure

The present study was approved by the Institutional Review Board of the Clinical Psychology department of the University of Amsterdam (2014-CP-3909) and conducted on eight consecutive days. On Day 1, participants were invited to the laboratory of the University of Amsterdam. After obtaining informed consent, participants filled in the self-report measures (LSAS-SR, FNE-B) on a computer. Next, participants completed the AAT practice directly followed by the AAT assessment. Before the succeeding virtual reality BAT, the experimenter assisted participants to put on the HMD and a microphone that were connected to a laptop. Then, participants entered the virtual situations (first the bus stop and then the language class) and rated their maximum anxiety level (virtual reality SUD) after each virtual situation. Then, the in vivo BAT followed and participants rated their maximum anxiety level afterward (in vivo SUD). Finally, participants were thanked for their participation on Day 1. On Days 2–8, participants received an email containing a link to an online self-report battery (SSA, SMEA, DES), which they completed at home between 06.00 and 12.00 p.m. We also sent one reminder per email and one reminder per SMS in case participants had not filled in the questionnaires until 10 p.m. Participants received the partial course credit after they filled in the questionnaire on Day 8.

### Statistical analyses

To investigate prospective predictors of daily social anxiety, experiential avoidance, and the experience of negative social events, we applied a step-wise procedure for each of the three outcome variables using IBM SPSS Statistics 24.0 (IBM, Armonk, NY, USA). This approach has been used in previous studies with large numbers of predictors and relative small sample sizes (Amir, Taylor & Donohue, 2011; Fournier et al., 2009; Kuckertz et al., 2015). We used this step-wise procedure in order to prevent the rejection of a possible predictor because of the sample size. Accordingly, we investigated the predictive value of the predictors of each domain separately before entering significant predictors to a final model. In Step 1, a separate regression model was calculated using the Enter-method for each of the three domains (self-report questionnaire, BAT, AAT) including all predictors. In Step 2, all predictors with a significance value of *p* < 0.20 in Step 1 were entered into a final model (Fournier et al., 2009). Here, a significance value of *p* < 0.05 was applied. Squared semipartial correlations (*sr*^2^) were calculated for all predictors to indicate the unique contribution of each predictor to a model. Data of one participant were missing for the three outcome variables (SSA, SMEA, DES) on Day 6 and of two participants for the AAT. Missing data were replaced with the group mean of each variable, respectively. All assumptions for multiple regression were met. See Table 1 for means and standard deviations of all variables.

**Table 1:** Means, standard deviations, and correlations.

	LSAS-SR	FNE-B	Virtual reality SUDs	In vivo SUDs	AAT angry faces	AAT disgusted faces	SSA	DES	SMEA
LSAS-SR		0.532**	0.576**	0.379**	−0.162	0.094	0.506**	0.127	0.474**
FNE-B			0.201	0.107	−0.087	0.009	0.712**	0.251*	0.511**
Virtual reality SUDs				0.464**	−0.006	−0.104	0.332*	0.108	0.221
In vivo SUDs					−0.153	−0.071	0.290*	0.253*	0.228
AAT angry faces						0.058	−0.201	−0.153	−0.117
AAT disgusted faces							0.052	0.166	0.102
SSA								0.534**	0.760**
DES									0.492**
SMEA									
Means (*SD*s)	37.16 (17.11)	23.10 (10.47)	3.65 (1.89)	6.21 (2.17)	5.39 (42.21)	1.17 (46.69)	13.91 (4.96)	17.24 (11.60)	2.42 (1.04)

**Notes:**

AAT, approach-avoidance task; FNE-B, fear of negative evaluation scale-brief form; DES, daily event survey; LSAS-SR, Liebowitz social anxiety scale-self-report; SMEA, state measure of experiential avoidance; SSA, state social anxiety questionnaire; SUD, subjective units of distress.

* *p* < 0.05;

** *p* < 0.01.

## Results

### Social anxiety

Correlational analyses revealed that scores of social anxiety in everyday life, as measured with the SSA, were significantly associated with LSAS-SR, FNE-B, virtual reality SUDs, and in vivo SUDs. This indicates that participants who reported higher fear and avoidance in social situations, fear of negative evaluation, and maximum anxiety levels during the virtual reality and in vivo BAT, reported higher social anxiety in daily life activities (see Table 1). To investigate possible predictors of everyday social anxiety, first, separate regression models were calculated for each of the three domains (Step 1). Both self-report questionnaires (Domain 1) yielded *p*-values below the threshold value of *p* < 0.20 in Step 1 (LSAS-SR: *β* = 0.18, *p* = 0.099, *sr*^2^ = 0.02 and FNE-B: *β* = 0.62, *p* < 0.001, *sr*^2^ = 0.27). Regarding behavioral assessments tasks (Domain 2), maximum anxiety levels during the virtual reality BAT were retained in the final model (virtual reality SUD: *β* = 0.25, *p* = 0.070, *sr*^2^ = 0.05) because the *p*-value was below the threshold value of *p* < 0.20 in Step 1. This was not the case for the *p*-value of the in vivo BAT (in vivo SUD: *β* = 0.17, *p* = 0.210, *sr*^2^ = 0.02) and therefore, maximum anxiety levels during the in vivo BAT were not entered to the final model. For the AAT (Domain 3), RTs for angry faces were retained in the final model (AAT angry: *β* = −0.21, *p* = 0.113, *sr*^2^ = −0.04) because of a *p*-value below the threshold value of *p* < 0.20 in Step 1. RTs for disgusted faces were not entered into the final model (AAT disgusted: *β* = 0.06, *p* = 0.615, *sr*^2^ = 0.00).

When the predictors retained from Step 1 were then entered into a final model (Step 2), only fear of negative evaluation (*β* = 0.65, *p* < 0.001, *sr*^2^ = 0.29) was significant and the final model explained 57% of the variance in SSA scores. Fear and avoidance in social situations (LSAS-SR: *β* = 0.04, *p* = 0.779, *sr*^2^ = 0.00), RTs for angry faces during the AAT (AAT angry: *β* = −0.14, *p* = 0.126, *sr*^2^ = −0.02), and maximum anxiety levels during the virtual reality BAT (virtual reality SUD: *β* = 0.18, *p* = 0.101, *sr*^2^ = 0.05) were no significant predictors of SSA scores in the final model, although the latter was approaching significance (see Table 2A for regression equations).

**Table 2:** Regression equations with (A) social anxiety (SSA), (B) experiential avoidance (SMEA), and (C) negative social events (DES) as outcome variables.

	*B*	Confidence interval		SE *B*	β	*t*	*p*	*sr*^2^	*R*^2^	*p*
	**	Lower	Upper							
**(A) SSA**										
Domain 1: questionnaires									0.53	<0.001
LSAS-SR	0.05	−0.01	0.11	0.03	0.18	1.68	**0.099**	0.02		
FNE-B	0.29	0.19	0.39	0.05	0.62	5.86	**0.000**	0.27		
Domain 2: behavioral assessment tasks									0.13	0.014
Virtual reality SUDs	0.66	−0.06	1.38	0.36	0.25	1.84	**0.070**	0.05		
In vivo SUDs	0.40	−0.23	1.02	0.31	0.17	1.27	0.210	0.02		
Domain 3: approach-avoidance task									0.05	0.259
Angry faces	−0.02	−0.05	0.01	0.02	−0.21	−1.61	**0.113**	−0.04		
Disgusted faces	0.01	−0.02	0.03	0.01	0.06	0.51	0.615	0.00		
Final model									0.57	<0.001
LSAS-SR	0.01	−0.06	0.08	0.04	0.04	0.28	0.779	0.00		
FNE-B	0.31	0.21	0.40	0.05	0.65	6.20	**0.000**	0.29		
Virtual reality SUDs	0.48	−0.10	1.05	0.29	0.18	1.67	0.101	0.05		
Angry	−0.02	−0.04	0.01	0.01	−0.14	−1.55	0.126	−0.02		
**(B) SMEA**										
Domain 1: questionnaires									0.32	<0.001
LSAS-SR	0.02	0.00	0.03	0.01	0.28	2.22	**0.031**	0.06		
FNE-B	0.04	0.01	0.06	0.01	0.36	2.85	**0.006**	0.09		
Domain 2: behavioral assessment tasks									0.07	0.121
Virtual reality SUDs	0.08	−0.08	0.24	0.08	0.15	1.04	0.304	0.02		
In vivo SUDs	0.08	−0.06	0.21	0.07	0.16	1.13	0.264	0.02		
Domain 3: approach-avoidance task									0.03	0.464
Angry faces	−0.00	−0.01	0.00	0.00	−0.12	−0.96	0.341	−0.02		
Disgusted faces	0.00	−0.00	0.00	0.00	0.11	0.85	0.398	0.01		
Final model									0.32	<0.001
LSAS-SR	0.02	0.00	0.03	0.01	0.28	2.22	**0.031**	0.06		
FNE-B	0.04	0.01	0.06	0.01	0.36	2.85	**0.006**	0.09		
**(C) DES**										
Domain 1: questionnaires									0.06	0.146
LSAS-SR	−0.01	−0.21	0.20	0.10	−0.01	−0.06	0.951	−0.00		
FNE-B	0.28	−0.05	0.61	0.17	0.26	1.72	**0.091**	0.05		
Domain 2: behavioral assessment tasks									0.06	0.142
Virtual reality SUDs	−0.08	−1.83	1.67	0.87	−0.01	−0.09	0.930	−0.00		
In vivo SUDs	1.39	−0.14	2.91	0.76	0.26	1.82	**0.074**	0.05		
Domain 3: approach-avoidance task									0.05	0.194
Angry faces	−0.05	−0.12	0.03	0.04	−0.16	−1.29	0.202	−0.03		
Disgusted faces	0.04	−0.02	0.11	0.03	0.18	1.38	**0.172**	0.03		
Final model									0.15	0.025
FNE-B	0.25	−0.02	0.52	0.14	0.22	1.84	0.072	0.05		
In vivo SUDs	1.30	−0.02	2.61	0.66	0.24	1.98	0.053	0.06		
Disgusted	0.05	−0.02	0.11	0.03	0.18	1.49	0.142	0.03		

**Notes:**

AAT, approach-avoidance task; FNE-B, fear of negative evaluation scale-brief form; BAT, behavior assessment task; DES, daily event survey; LSAS-SR, Liebowitz social anxiety scale-self-report; SMEA, state measure of experiential avoidance; SSA, state social anxiety questionnaire; SUD, subjective units of distress.

Significant *p*-values (*p* < 0.20 in Step 1, *p* < 0.05 in Step 2) are marked in bold.

### Experiential avoidance

Correlational analyses revealed that scores of experiential avoidance assessed in everyday life were significantly associated with LSAS-SR and FNE-B scores, indicating that participants who reported higher fear and avoidance in social situations and fear of negative evaluation in the laboratory, reported higher experiential avoidance in everyday life (see Table 1). To determine possible predictors of experiential avoidance, we ran a separate regression model for each of the three domains, with SMEA as outcome variable (Step 1). For self-report questionnaires (Domain 1), both fear and avoidance in social situations (LSAS-SR: *β* = 0.28, *p* = 0.031, *sr*^2^ = 0.06) and fear of negative evaluation (FNE-B: *β* = 0.36, *p* = 0.006, *sr*^2^ = 0.09) showed *p*-values below the threshold value of *p* < 0.20 and could be retained in the final model. Results for behavioral avoidance tasks (Domain 2) revealed that neither maximum anxiety levels during the virtual reality BAT (SUD virtual reality: *β* = 0.15, *p* = 0.304, *sr*^2^ = 0.02) nor during the in vivo BAT (in vivo SUD: *β* = 0.16, *p* = 0.264, *sr*^2^ = 0.02) predicted SMEA scores. Results for the AAT (Domain 3) showed that neither RTs for angry faces (AAT angry: *β* = −0.12, *p* = 0.341, *sr*^2^ = −0.02) nor disgusted faces (AAT disgusted: *β* = 0.11, *p* = 0.398, *sr*^2^ = 0.01) predicted SMEA scores in Step 1. Therefore, the final model comprised only LSAS-SR and FNE-B and was hence similar to the individual model of Domain 1, explaining 33% of the variance in SMEA scores (see Table 2B for regression equations).

### Negative social events

Correlational analyses showed that the experience of everyday negative social events measured using the DES, significantly correlated with FNE-B scores and in vivo SUDs. This indicates that participants who reported higher fear of negative evaluations and maximum anxiety levels during the in vivo BAT in the laboratory, reported a higher percentage of negative social events in everyday life (see Table 1). Separate multiple regression analyses with DES scores as outcome variable were conducted for the three domains to determine possible predictors of the experience of negative social events (Step 1). Results for self-report questionnaires (Domain 1) revealed that fear of negative evaluation (FNE-B: *β* = 0.26, *p* = 0.091, *sr*^2^ = 0.05) reached a *p*-value below the threshold (*p* < 0.20) and was hence retained in the final model. Fear and avoidance was not entered into the final model (LSAS-SR: *β* = −0.01, *p* = 0.951, *sr*^2^ = −0.00). Maximum anxiety levels during BATs (Domain 2) were retained in the final model for the in vivo BAT only because of a *p*-value below the threshold value of *p* < 0.20 in Step 1 (in vivo SUD: *β* = 0.26, *p* = 0.074, *sr*^2^ = 0.05). Maximum anxiety levels during the virtual reality BAT were not entered into the final model (virtual reality SUD: *β* = −0.01, *p* = 0.930, *sr*^2^ = −0.00). For the AAT (Domain 3), only RTs for disgusted faces were retained in the final model (AAT disgusted: *β* = 0.18, *p* = 0.172, *sr*^2^ = 0.03) because of the *p*-value below the threshold value of *p* < 0.20 in Step 1. The *p*-value for RTs for angry faces exceeded this threshold (AAT angry: *β* = −0.16, *p* = 0.202, *sr*^2^ = −0.03).

However, although *p*-values of fear of negative evaluation (FNE-B: *β* = 0.22, *p* = 0.072, *sr*^2^ = 0.05) and maximum anxiety levels during the in vivo BAT (SUD in vivo: *β* = 0.24, *p* = 0.053, *sr*^2^ = 0.06) approached significance (*p* < 0.05 in Step 2), none of the variables in the final model significantly predicted the experience of negative social events. The final model significantly fitted the data (*R*^2^ = 0.15, *p* = 0.025). See Table 2C for regression equations.

## Discussion

The present study aimed to investigate the potential of self-report questionnaires, in vivo and virtual reality BATs, and approach-avoidance biases as measured by the AAT for predicting social anxiety, experiential avoidance, and the experience of negative social events in everyday life. The results revealed that the self-report questionnaire assessing fear of negative evaluation predicted both social anxiety and experiential avoidance in the following week. The self-report questionnaire assessing fear and avoidance in social situations predicted everyday experiential avoidance. Neither approach-avoidance bias for angry faces and disgusted faces during the AAT nor the in vivo and virtual reality BATs significantly predicted any of the three outcome variables.

Self-report questionnaires are commonly used in the assessment of social anxiety and our results predominantly support their value as assessment tools. Our results particularly support self-report of fear of negative evaluation as a useful assessment tool for social anxiety because it predicted both everyday social anxiety and experiential avoidance. As indicated by significant positive correlations, individuals who reported higher fear of negative evaluation, also reported higher social anxiety, experiential avoidance, and more negative social events in everyday life. The questionnaire measuring fear and avoidance in social situations predicted experiential avoidance but not social anxiety in everyday life.

A reason why fear of negative evaluation was a predictor of social anxiety in daily life whereas fear and avoidance in social situations was not, might be, that the former might better tap the core feature of social anxiety. The fear of negative evaluation is considered to be a fundamental component of social anxiety, crucial for development and maintenance of excessive anxiety (Clark & Wells, 1995; Rapee & Heimberg, 1997), and is part of the criteria for social anxiety disorder (DSM-V; American Psychiatric Association, 2013). Avoidance on the other hand is not prerequisite for a diagnosis as long situations are endured with intense anxiety (American Psychiatric Association, 2013). Although neither of the self-report questionnaires predicted the experience of negative social events (fear of negative evaluation approached significance), both were significantly associated with the experience of negative social events, indicating that the more fear of negative evaluation and fear and avoidance in past social situations individuals reported, the more negative social events relative to positive events, they experienced in daily life. This is in line with previous research that found social anxiety to be associated with increased experiences of negative events (Gilboa-Schechtman, Franklin & Foa, 2000).

The results of the present study did not support BATs as assessment tools for social anxiety. Although maximum anxiety levels during both the in vivo and the virtual reality BAT were associated with everyday social anxiety, indicating that individuals with higher maximum anxiety during both BATs reported higher social anxiety in daily life, neither proved to be a significant predictor of everyday social anxiety, experiential avoidance, or experience of negative social events. Yet the in vivo BAT approached significance for the prediction of negative social events. Therefore, future research is needed to address a possible power issue by replicating these findings with a greater sample size. Furthermore, the in vivo BAT comprised an impromptu speech and therefore it might not have triggered anxiety in individuals with predominantly other social fears than fear of public speaking. This is particularly important for individuals who might primarily fear verbal exchange in social situations and the BAT did not comprise verbal interaction. In contrast, the virtual reality BAT included social interaction in form of virtual encounters and conversation, and in line with this, the virtual reality BAT was approaching statistical significance as a predictor for social anxiety. Technical improvements regarding the virtual reality system used might contribute to the predictive value of the virtual reality BAT in the future. The virtual humans in the present study could not show different facial expressions. However, varying facial expressions (e.g., angry faces) might help to target fear of negative evaluation, a core feature of social anxiety and might contribute to the experience of presence, the feeling of being present in the virtual world. Additionally, using questions related to personal topics might also help to increase the experience of social anxiety during the in vivo and virtual reality BAT. Future research needs to investigate whether expanding the in vivo BAT regarding social situations that include verbal interaction might improve its predictive value. However, considering that both in vivo and virtual reality BAT are time and cost consuming, the added value of BATs compared to self-report questionnaires can be questioned.

Contrary to previous studies that found elevated automatic avoidance tendencies for angry faces in social anxiety (Heuer, Rinck & Becker, 2007; Roelofs et al., 2010), the results of this study do not indicate that implicit action tendencies predict social anxiety in everyday life. Neither approach-avoidance bias for angry nor disgusted faces predicted social anxiety, experiential avoidance, and negative social events in the following week. These null-findings might be explained, however, by task characteristics. During the AAT, participants responded to stimuli based on color categorization instead of affective valence. Phaf et al. (2014) reported in their meta-analysis that non-significant effects were consistently associated with task instructions that did not demand “conscious appraisals of the affective valence of stimuli.” In future studies, the power of the AAT might be increased by using emotional categorization. At this point, our results do not support the potential of the AAT as a standardized assessment task for social anxiety.

A limitation of the present study was that the dependent variables were assessed using self-report measures. The results for the self-report questionnaires for fear of negative evaluation and fear and avoidance in social situations might hence partly be explained by shared measurement variance. In future studies, these results need to be replicated using measures other than self-reporting, such as behavioral assessments, physiological measures (also as an independent variable), or ratings by observers. Moreover, in the present study, all participants entered the two virtual scenarios in the virtual BAT in the same sequence. In future studies, the order of the virtual scenarios should be counterbalanced. Another limitation was the small sample size relative to the number of predictors. Future studies comprising a greater sample size are needed to replicate our findings and investigate effects that might not have reached significance in the present study due to the limited power. Additionally, the present study comprised a non-clinical sample, and therefore our results cannot be generalized to individuals with a diagnosis of social anxiety disorder. However, although participants were not formally diagnosed with social anxiety disorder in the current sample, average levels of fear and avoidance in social situations as assessed with the LSAS-SR (*M* = 37.16) exceeded the suggested cut-off score (i.e., 30) for social anxiety disorder (Mennin et al., 2002) and also approached (*M* = 23.10) the cut-off score of 25 for the FNE-B measuring fear of negative evaluation (Carleton et al., 2011). Future research needs to replicate our findings in clinical samples.

## Conclusions

We found self-report questionnaires measuring fear of negative evaluation and fear and avoidance in social situations to predict everyday experiential avoidance, and the former also everyday social anxiety. While our results instigate to question the value of BATs as assessment tools, they clearly support the use of self-report questionnaires in the assessment of social anxiety.

## Supplemental Information

10.7717/peerj.5441/supp-1 Supplemental Information 1 Self-report data and anxiety levels during the in vivo and virtual reality BAT.

10.7717/peerj.5441/supp-2 Supplemental Information 2 Data AAT.

10.7717/peerj.5441/supp-3 Supplemental Information 3 Questionnaires.

## Additional Information and Declarations

### Data Availability

The following information was supplied regarding data availability: The raw data are provided in the Supplemental Files.
