# Supplementary material for: Self-report questionnaires, behavioral assessment tasks, and an implicit behavior measure: do they predict social anxiety in everyday life?
Source: PeerJ. 2018 Aug 10;6:e5441. doi: 10.7717/peerj.5441 (PMC6089206; doi:10.7717/peerj.5441)
Supplement: Supplemental Information 3 [file peerj-06-5441-s003.pdf]

Liebowitz Social Anxiety Scale (LSAS)

Vink onder Angst/vrees en Vermijding het nummer aan dat het best overeenkomt met hoe u zich de afgelopen week voelde. Let er op dat u alle vragen beantwoordt.

Hoeveel angst of vrees veroorzaken de volgende situaties bij u? Hoezeer tracht u ze te vermijden? Angst/vrees: 0 = helemaal niet, 1 = een beetje, 2 = nogal, 3 = heel erg; Vermijding: 0 = nooit, 1 = soms, 2 = vaak 3 = bijna altijd.

	**Angst/vrees**	**Vermijding**
	**0 1 2 3**	**0 1 2 3**
1.Telefoneren in gezelschap. (P)	□ □ □ □	□ □ □ □
2.Deelnemen aan een activiteit in een kleine groep. (P)	□ □ □ □	□ □ □ □
3.Eten in gezelschap. (P)	□ □ □ □	□ □ □ □
4.Drinken in gezelschap. (P)	□ □ □ □	□ □ □ □
5.Met belangrijke mensen praten. (S)	□ □ □ □	□ □ □ □
6.In het openbaar spreken of iets doen. (P)	□ □ □ □	□ □ □ □
7.Naar een feestje gaan. (S)	□ □ □ □	□ □ □ □
8.Werken terwijl er een ander toekijkt of u observeert. (P)	□ □ □ □	□ □ □ □
9.Schrijven terwijl een ander toekijkt. (P)	□ □ □ □	□ □ □ □
10.Een redelijk onbekend iemand opbellen. (S)	□ □ □ □	□ □ □ □
11.Met oppervlakkige kennissen praten. (S)	□ □ □ □	□ □ □ □
12.Ontmoeten van vreemden. (S)	□ □ □ □	□ □ □ □
13.Een openbaar toilet of urinoir gebruiken. (P)	□ □ □ □	□ □ □ □
14.Een kamer met mensen binnengaan. (P)	□ □ □ □	□ □ □ □
15.In het middelpunt van de aandacht staan. (S)	□ □ □ □	□ □ □ □
16. Zonder voorbereiding iets zeggen in een vergadering.(P)	□ □ □ □	□ □ □ □
17.Een examen of een test doen. (P)	□ □ □ □	□ □ □ □
18.Kritiek geven op iemand die u niet zo goed kent. (S)	□ □ □ □	□ □ □ □
19.Mensen die u niet zo goed kent aankijken. (S)	□ □ □ □	□ □ □ □
20.In een groep mensen van iets verslag uitbrengen. (P)	□ □ □ □	□ □ □ □
21. Een afspraakje maken met iemand of iemand'versieren'. (P)	□ □ □ □	□ □ □ □
22. Spullen naar een winkel terugbrengen en uw geld terugvragen. (S)	□ □ □ □	□ □ □ □
23.Een feestje geven. (S)	□ □ □ □	□ □ □ □
24.Een opdringerige verkoper aan de deur afwimpelen. (S)	□ □ □ □	□ □ □ □

Ppnr: …………

BFNE-II-NL

Lees elk van de volgende uitspraken aandachtig door en geef aan hoe goed zo'n uitspraak bij u past: 0 = past helemaal niet bij mij, 1 = past een beetje bij mij, 2 = past redelijk bij mij, 3 = past goed bij mij, 4 = past heel erg goed bij mij.

	Past helemaal niet bij mij<br>0	Past een beetje bij mij<br>1	Past redelijk bij mij<br>2	Past goed bij mij<br>3	Past heel erg goed bij mij<br>4
1. Soms denk ik dat ik me teveel zorgen maak over wat andere mensen van mij vinden.	0	1	2	3	4
2. Ik maak me zorgen over welke indruk ik op mensen maak.	0	1	2	3	4
3. Ik ben bang dat anderen iets op mij aan te merken zullen hebben.	0	1	2	3	4
4. Ik maak me zorgen over hoe andere mensen over mij denken.	0	1	2	3	4
5. Als ik met iemand praat maak ik me zorgen over wat diegene van mij denkt.	0	1	2	3	4
6. Ik ben bang dat anderen mij zullen afkeuren.	0	1	2	3	4
7. Ik maak me doorgaans zorgen over de indruk die ik maak.	0	1	2	3	4
8. Ik ben vaak bang dat anderen mijn tekortkomingen opmerken.	0	1	2	3	4
9. Ik maak me zorgen over wat anderen van mij denken, ook al weet ik dat het niets uitmaakt.	0	1	2	3	4
10. Ik maak me er druk om als mensen een ongunstige indruk van mij krijgen.	0	1	2	3	4
11. Ik maak me er vaak zorgen over dat ik het verkeerde zal doen of zeggen.	0	1	2	3	4
12. Als ik weet dat iemand mij beoordeelt, zit me dat doorgaans dwars.	0	1	2	3	4

Geautoriseerde Nederlandse vertaling © R. Cieraad & P.J. de Jong (2007). Rijksuniversiteit Groningen.

State Social Anxiety
Kashdan en Steger (2006)

Lees elk van de volgende uitspraken goed door en geef aan in welke mate zo’n uitspraak van toepassing is op de dag die je vandaag hebt gehad:
0 (helemaal niet), 1 (een beetje), 2 (matig), 3 (?) en 4 (heel erg).

1. Ik maakte mij zorgen om wat andere mensen van mij zouden denken.
2. Ik was bang dat andere mensen mijn tekortkomingen zouden opmerken.
3. Ik was bang dat andere mij zouden afkeuren.
4. Ik maakte mij zorgen dat ik dingen verkeerd zou zeggen of doen.
5. Wanneer ik met iemand aan het praten was, maakte ik mij druk om wat ze van mij zouden denken.
6. Ik voelde mij ongemakkelijk en beschaamd toen ik in het middelpunt van de belangstelling stond.
7. Ik vond het moeilijk om met mensen om te gaan.

1. I worried about what other people thought of me.
2. I was afraid other people noticed my shortcomings.
3. I was afraid that others did not approve of me.
4. I was worried that I would say or do the wrong things.
5. When I was talking to someone, I was worried about what they were thinking of me.
6. I felt uncomfortable and embarrassed when I was the center of attention.
7. I found it hard to interact with people.

State Measure of Experiential Avoidance – Daily diary version

- Kashdan, Goodman, Machell, Kleiman, Monfort, Ciarrochi & Nezlek, 2014

Lees elk van de volgende vragen goed door en beantwoord de vragen voor de dag die je vandaag hebt gehad. Beantwoord op een schaal van 1 = Helemaal niet, 4 = matig tot 7 = heel erg.

How upset and bothered were you about anxiety-related feelings or thoughts?	1 2 3 4 5 6 7
How much did you try to hide and/or conceal your anxiety from others?	1 2 3 4 5 6 7
How much did you try to control your anxiety-related feelings or thoughts?	1 2 3 4 5 6 7
To what degree did you give up saying or doing what you like (or mattered to you) in order to control and manage your anxiety?	1 2 3 4 5 6 7

Hoe van streek was je over en hoeveel last had je van angst gerelateerde gevoelens en gedachtes?	1 2 3 4 5 6 7
Hoe erg probeerde je je angst te verbergen en/of weg te stoppen voor andere?	1 2 3 4 5 6 7
Hoe erg probeerde je je angst gerelateerde gevoelens en gedachtes te controleren?	1 2 3 4 5 6 7
In welke mate heb je het doen of zeggen wat je leuk vind (of wat belangrijk voor je is) opgegeven om zo je angst te controleren en er mee om te kunnen gaan?	1 2 3 4 5 6 7

Daily events survey

Zijn de volgende gebeurtenissen/situaties gedurende de dag voorgekomen?

⇒ Beantwoord de vragen met JA of NEE.

Positive social events

1. Had especially good interactions with friend(s) or acquaintances .

- Had vooral goede interacties met vrienden of kennissen.

7. Went out socializing with friends/date (e.g., party, dance club) .

- Ging uit met vrienden/date (bijvoorbeeld een feest of uitgaansgelegenheid).

9. Had especially good interactions with my steady date .

- Had vooral goede interacties met mijn vaste date.

13. Did something special for a friend/steady date that was appreciated.

- Deed iets special voor een vriend/vaste date dat werd gewaardeerd.

14. Flirted with someone or arranged a date.

- Heb geflirt met iemand of regelde een date.

19. Went out to eat with a friend/date.

- Ging uiteten met een vriend/vaste date.

23. Had other type of pleasant event (not listed above) with friends, family, or date.

- Had andere soorten plezierige gebeurtenissen (hierboven niet genoemd) met vrienden, familie of vaste date.

Negative social events

4. Did something awkward or embarrassing in a social situation.

- Deed iets gênants of beschamends in een sociale situatie.

5. Was excluded or left out by my group of friends.

- Werd buitengesloten of … door mijn groep vrienden/vriendinnen.

11. A disagreement with a close friend or steady date was left unresolved.

- Een meningsverschil met een goede vriend/vriendin of vaste date werd niet opgelost.

16. Got along poorly with peers (e.g., classmates, co-workers, roommates).

- Kon slecht opschieten met leeftijdsgenoten (bijvoorbeeld klasgenoten, collega’s, huisgenoten).

22. Had plans fall through to spend time with someone special.

- Plannen om tijd door te brengen met een special persoon gingen niet door.

24. Had other type of unpleasant event (not listed above) with friends, family, or date.

- Had andere soorten onplezierige gebeurtenissen (hierboven niet genoemd) met vrienden, familie of vaste date.
